# Seasonally Varying Effects of Improved Water, Sanitation, and Handwashing Interventions on *Giardia* Infection in Bangladesh

**DOI:** 10.1093/ofid/ofag430

**Published:** 2026-07-16

**Authors:** Pearl Anne Ante-Testard, Francois Rerolle, Mahbubur Rahman, Rashidul Haque, Shimul Das, Sarker Masud Parvez, Ayse Ercumen, Audrie Lin, Stephen P Luby, Tarik Benmarhnia, Benjamin F Arnold

**Affiliations:** Francis I. Proctor Foundation and the Department of Ophthalmology, University of California, San Francisco, San Francisco, California, USA; Francis I. Proctor Foundation and the Department of Ophthalmology, University of California, San Francisco, San Francisco, California, USA; Environmental Health and WASH, Health System and Population Studies Division, International Centre for Diarrhoeal Disease Research, Dhaka, Bangladesh; Environmental Health and WASH, Health System and Population Studies Division, International Centre for Diarrhoeal Disease Research, Dhaka, Bangladesh; Environmental Health and WASH, Health System and Population Studies Division, International Centre for Diarrhoeal Disease Research, Dhaka, Bangladesh; Environmental Health and WASH, Health System and Population Studies Division, International Centre for Diarrhoeal Disease Research, Dhaka, Bangladesh; School of Public Health and Social Work, Queensland University of Technology, Brisbane, Queensland, Australia; Department of Forestry and Environmental Resources, North Carolina State University, Raleigh, North Carolina, USA; Department of Microbiology and Environmental Toxicology, University of California, Santa Cruz, Santa Cruz, California, USA; Division of Infectious Diseases and Geographic Medicine, Stanford University, Stanford, California, USA; Scripps Institution of Oceanography, University of California, San Diego, La Jolla, California, USA; Irset Institut de Recherche en Santé, Environnement et Travail, UMR-S 1085, Inserm, University of Rennes, EHESP, Rennes, France; Francis I. Proctor Foundation and the Department of Ophthalmology, University of California, San Francisco, San Francisco, California, USA

**Keywords:** Bangladesh, climate, effect modification, Giardia, WASH

## Abstract

**Background:**

*Giardia* is the most common enteric parasite among children in low-resource settings, causing diarrhea and leading to prolonged infection or asymptomatic carriage. We assessed whether the effect of water, sanitation, and handwashing (WSH) interventions on *Giardia* infection among rural Bangladeshi children varies with seasonal conditions.

**Methods:**

We conducted a secondary analysis of the WASH Benefits Bangladesh cluster-randomized trial, with 450 clusters assigned to 4 arms in a 2 × 2 factorial design (WSH: WSH, WSH + Nutrition; no WSH: Control, Nutrition). *Giardia* infection was measured by multiplex real-time polymerase chain reaction in stool samples after 2 years of intervention. Effects were estimated by marginal treatment and assessed for heterogeneity by season when *Giardia* was measured. We also assessed heterogeneity by cumulative exposure to dry and monsoon seasons from birth to measurement age to estimate cumulative seasonal exposure history.

**Results:**

*Giardia* prevalence, measured among 2773 children (median age: 30 months, range: 22–38 months), was higher in the dry seasons (32%) than in the monsoon (21%). Improved WSH was associated with a consistent 20% relative reduction and a modest absolute reduction that was slightly greater during the dry season (−6.1%; 95% confidence interval: −10.1 to −2.1), although interaction test did not show strong evidence of effect modification by season. Prevalence remained lower in the WSH group across increasing dry season exposure, with the largest differences among children with more than 17 months of dry-season exposure.

**Conclusions:**

We demonstrate how WSH provides resilience to seasonal variation in infection risk and mitigates climate-driven, seasonally varying *Giardia* transmission.

## Teaser

We investigated whether the effect of water, sanitation, and handwashing (WSH) interventions on *Giardia* infection among rural Bangladeshi children varies with seasonal conditions. We found that *Giardia* risk varied seasonally, and improved WSH mitigates climate-driven, seasonally varying transmission.

*Giardia duodenalis* (also known as *G. lamblia* or *G. intestinalis*) is one of the most prevalent enteric parasites among children living in low-resource settings, where it contributes to both symptomatic and asymptomatic infections [1, 2]. Globally, *Giardia* is a leading cause of diarrheal illness in children, following rotavirus and *Cryptosporidium* species, with >300 million cases reported annually [3]. In areas with inadequate sanitation, giardiasis prevalence can reach 20% to 40%, with the highest infection rates observed in children younger than age 5 years [1]. Despite its high burden, it remains one of the most neglected intestinal parasites [4]. Transmission occurs through waterborne, foodborne, or person-to-person pathway. Although *Giardia* can cause acute diarrhea, children infected are often asymptomatic [4] with prolonged carriage that may impair growth and development in early childhood. *Giardia* can be prevented through water, sanitation, and handwashing (WSH) interventions, which disrupt fecal-oral transmission pathways by ensuring safe containment and disposal of feces and by promoting hand hygiene to reduce contamination from caregivers' hands and food [5–7].

Seasonal variation plays an important role in the transmission of enteric pathogens, including *Giardia*, but the nature of these relationships differs across settings. In urban France, rainfall and *Giardia* concentrations were weakly positively associated [8]. In rural Bangladesh, a previous analysis of the WASH Benefits Bangladesh trial reported lower *Giardia* prevalence following weeks with above-median precipitation [9], whereas a study from Mexico showed that *Giardia* incidence often peaks in the spring and summer months, particularly in warm and humid climates [10]. Seasonality may also influence interventions effects of WSH, which are a key strategy in reducing diarrhea morbidity and mortality [11, 12]. In the WASH Benefits Bangladesh trial, reductions in all-cause diarrhea due to WSH interventions were greatest during the monsoon season [13, 14]. In addition, although improved WSH reduced *Giardia* infection in the WASH Benefits trial [5], its effectiveness in mitigating climate-driven *Giardia* infection remains unclear.

Here, we assess whether the impact of WSH interventions on *Giardia* infection differs between dry and monsoon seasons, reflecting the influence of climate-sensitive exposures. We hypothesized that effect of improved WSH on *Giardia* infection could vary by season during periods of heightened environmental exposure when transmission risk is likely to be elevated.

## METHODS

### Study Population

We conducted a secondary analysis of the WASH Benefits Bangladesh cluster randomized trial in 4 rural districts in Bangladesh—Gazipur, Mymensingh, Tangail, and Kishoreganj. The study design and rationale [15] and primary outcomes [11] were previously published.

The trial enrolled pregnant women between 31 May 2012 and 7 July 2013 who self-reported being in their first or second trimester and measured outcomes in their newborn (index) children for 2 years. The enabling environment and behavioral intervention packages were developed in the target populations during the 2 years preceding the trials [15]. Groups of 8 mothers were organized into clusters, with each cluster spaced approximately 1 km apart to minimize the risk of spillover. We focused on index children in this analysis.

### Study Design

A total of 720 clusters, grouped into blocks of 8 geographically matched clusters, were randomly assigned to either a double-sized control group (C) or 1 of 6 intervention groups: improved drinking water (W), sanitation (S), handwashing (H), nutrition (N), combined water, sanitation, and handwashing (WSH), and combined WSH and nutrition (WSH + N). Lipid-based nutrient supplementation ended when children reached 24 months of age, whereas WSH interventions continued until 36 months after intervention initiation [16]. Community health promoters visited intervention households at least weekly during the first 6 months, and at least biweekly thereafter to encourage recommended behaviors. Control households never received the interventions nor promoter visits.

This analysis focused on children from clusters that received the combined WSH interventions (WSH and WSH + N) and those from the control group (n = 360 clusters), and Nutrition arm, excluding clusters assigned to other single interventions to satisfy the consistency assumption in causal inference [17]. We analyzed the interventions in a 2 × 2 factorial design (WSH: WSH and WSH + N, no WSH: N and control) with a focus on WSH intervention effects, as previous analyses found no evidence that the Nutrition intervention affected *Giardia* infection [5]. Adherence to combined interventions and nutrition was high and consistent over time, with objective measures exceeding 80% [11, 18]. Separately, objective fidelity assessment showed that intervention was being implemented as intended by the sixth month of assessment [19]. Feeding practices, including breastfeeding versus bottle feeding, may influence *Giardia* infection risk through exposure to contaminated water used for bottle washing or feeding. We did not account for feeding practices in the primary analysis because of data unavailability and because exclusive breastfeeding was common in early infancy and nearly all children would have been weaned by *Giardia* measurement. Use of treated water among index children was also high, with an uptake of >65% (range 66%–74%) [18]. However, because *Giardia* cysts are relatively chlorine-resistant, reduced exposure likely occurred through broader sanitation and hygiene pathways.

### Outcome

We measured *Giardia* infection status, coded as 1 for a positive test and 0 for a negative test, using multiplex real-time polymerase chain reaction (PCR), assessed ∼2.5 years after the intervention. Stool samples were collected from all birth cohort children, irrespective of diarrheal symptoms; therefore, our outcome includes both symptomatic and asymptomatic infections.

Trained staff distributed sterile fecal containers, instructed caregivers to collect samples from the next morning's defecation, and retrieved them the same day. Samples were transported on ice to a satellite laboratory of the icddr,b (Dhaka), stored at −80 °C, and later shipped on dry ice to icddr,b for analysis of *Giardia*. Genomic DNA was extracted from 200 mg of stool using the QIAamp Fast DNA Stool Mini Kit according to the manufacturer's instructions and eluted in 200 µL of AE buffer [5]. Protozoa were detected by multiplex real-time PCR following a previously published protocol [20], targeting the small subunit 18 seconds rRNA gene of *Giardia intestinalis* (62 bp, GenBank M54878). Samples were deemed positive to *Giardia* for cycle threshold (Ct) values <40 [5].

### Effect Modifiers

*Season*. We defined monsoon season as the months with elevated precipitation and persistent rainfall, where the rolling 5-day average was above 10 mm based on previous analyses of the trial from 2012 to 2016 (12 May-9 October 2012, 27 April-6 October 2013, 27 May-27 September 2014, 1 April-26 September 2015, and 12 May-26 June 2016) [9]. Months that fell outside monsoon seasons (coded as 0) were classified as dry season (coded as 1).

*Cumulative dry and monsoon months*. Asymptomatic carriage of *Giardia* is common, making it difficult to determine the exact timing of infection. Thus, we also used the cumulative months that a child had been exposed to the dry and monsoon period from birth up to their measurement age between 2012 and 2016 to estimate a child's cumulative seasonal exposure history.

### Statistical Analyses

The analysis was conducted by intention-to-treat and a complete case analysis. First, we conducted descriptive statistics of the study characteristics and effect modifiers between the WSH and no WSH groups.

Second, we estimated the effect of WSH on *Giardia* by binary season (dry vs monsoon) using a generalized linear model. We used a binomial family with identity link to estimate the absolute effects (Prevalence Difference: PD) and *Giardia* prevalence, and a log link to estimate relative effects (Prevalence Ratios: PR). We accounted for clustering at block level using robust standard errors. Because the analysis followed a 2 × 2 factorial design, we first tested for interaction between the WSH and Nutrition interventions. As no evidence of interaction was observed (additive interaction *P* = .91; multiplicative interaction *P* = .77), we proceeded with analysing effects at the margin—the effect of each intervention (WSH) was adjusted for the other intervention and any covariates and assumed that the effect of each intervention is uninfluenced by the presence or absence of the other [21], allowing us to estimate the effects of each intervention independently [22].

We calculated the PD, PR, and their standard errors [23] by estimating the marginal effect of WSH intervention on *Giardia* conditional on season and WSH (while adjusting for Nutrition). Pairwise contrasts comparing the WSH and no WSH groups were then estimated within each season stratum (dry and monsoon). We also estimated these effects using the linear combinations of the regression coefficients obtained from the regression models as a robustness check.

Third, the effect of WSH on *Giardia* prevalence was modeled as a smooth function of cumulative dry- and monsoon-month exposure using a generalized additive model [24], conditional on cumulative season exposure and WSH and adjusted for age, birth year, and Nutrition. Cumulative seasonal exposure is inherently related to time since intervention initiation and child birth year. Because time since intervention is also correlated with child age, we adjusted for both child age at measurement and birth year to account for variation in exposure duration and birth timing. Pointwise differences between WSH and no WSH smooths were estimated [25].

### Additional and Sensitivity Analyses

We estimated the effect of Nutrition compared with No Nutrition to complete the 2 × 2 factorial analysis. We conducted sensitivity analyses that excluded the first 6 months of life when calculating the cumulative number of dry and monsoon months each child had experienced, excluding the earliest period of life when children were less likely to interact with their environment [26] and consume complementary foods [27].

## RESULTS

### Study Population

A total of 2273 index children were included in the analysis, of whom 985 received any form of combined WSH intervention (WSH and WSH + N) and 1288 did not (Nutrition and Control) (Table 1). Baseline characteristics, index child sex, and potential effect modifiers were generally well balanced across study groups. Most of the children were born in 2013, accounting for nearly 90% of the participants, with fewer births in 2012 and 2014. Protozoan specimen collection was conducted in 2015 and 2016. Collection was nearly evenly distributed across 2015 and 2016, with slightly more children sampled in 2016. Overall, the children in different study groups were of similar age at the time of measurement, averaging about 2.5 years old. Most of the children were surveyed during the dry season compared to the monsoon season. The trial was conducted only in areas that were not at risk of being heavily flooded during seasonal monsoon, which may partially explain more data collection in the dry season.

**Table 1.** Study Characteristics and Summary of Effect Modifiers Between WSH and no WSH

	No WSH (N = 1288)	WSH (N = 985)
**Characteristics**		
**Sex**		
**Female**	645 (50.1%)	500 (50.8%)
**Male**	643 (49.9%)	485 (49.2%)
**Year of birth**		
**2012**	111 (8.6%)	86 (8.7%)
**2013**	1150 (89.3%)	875 (88.8%)
**2014**	27 (2.1%)	24 (2.4%)
**Stool sample collection**		
**2015**	575 (44.6%)	434 (44.1%)
**2016**	713 (55.4%)	551 (55.9%)
**Age in days** **Mean (SD)**	910 (57.1)	907 (55.8)
**Protozoa specimen**		
**Missing**	39 (3.0%)	33 (3.4%)
**Observed**	1249 (97.0%)	952 (96.6%)
**Effect modifiers**		
**Season**		
**Dry**	940 (73.0%)	690 (70.1%)
**Monsoon**	348 (27.0%)	295 (29.9%)
**Number of dry months** **Mean (SD)**	17.7 (1.99)	17.7 (2.02)
**Number of monsoon months** **Mean (SD)**	12.2 (1.83)	12.2 (1.79)

WSH: received any form of water, sanitation and handwashing interventions (WSH and WSH + N); no WSH: did not receive any form of WSH (control and Nutrition groups). SD: Standard deviation.

### Heterogeneity by Exposure to the Dry and Monsoon Seasons According to Birth Timing

**Figure 1.**
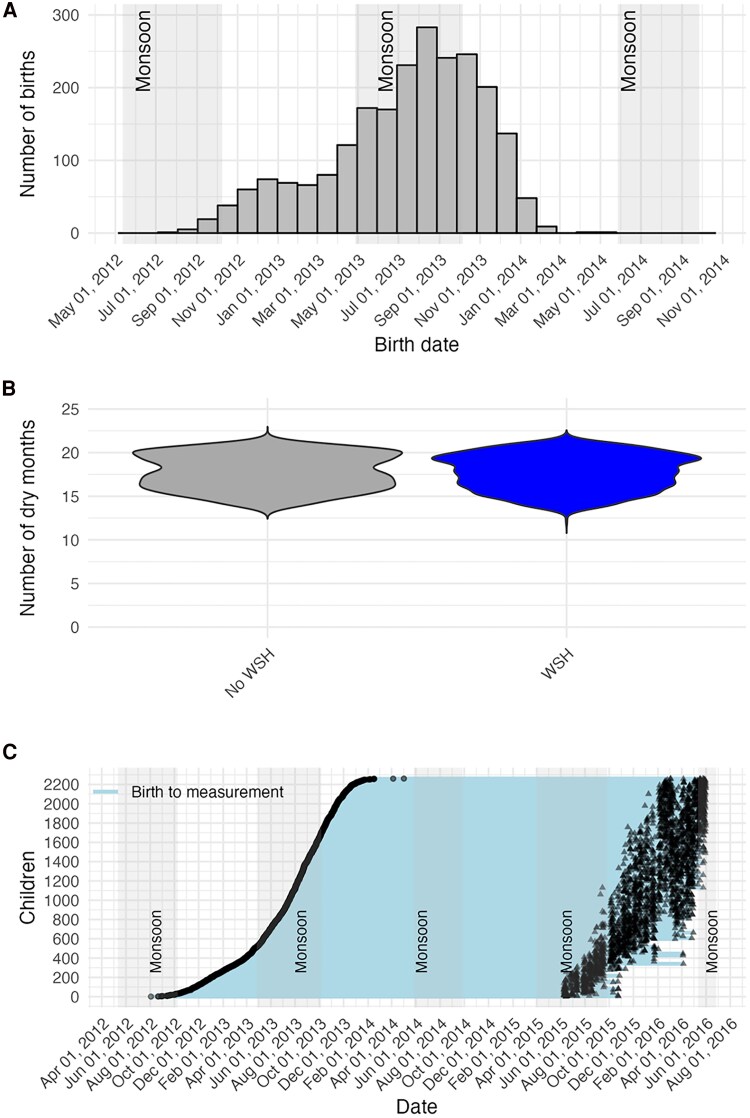
Children in the cohort experienced the dry and monsoon seasons from birth to the time of measurement. *A)* Birth dates of children included in the study with monsoon months shaded in gray. *B*) Distribution of the number of dry months by study groups. *C*) Timeline from a child's birth to measurement, with monsoon months shaded in gray to show the cumulative months of exposure to monsoon. Children are ordered by birth date.

Children were born between 3 August 2012 and 2 May 2014. Frequency of these births has occurred more during the monsoon period (Figure 1*A*). The cumulative number of dry months experienced by children was similar between the WSH and no WSH groups (Figure 1*B*). However, because births occurred at different times of the year, children's cumulative exposure to dry and monsoon seasons from birth to time of measurement varied except for those born in the same day (Figure 1*C*).

### Effect Modification by Season

**Figure 2.**
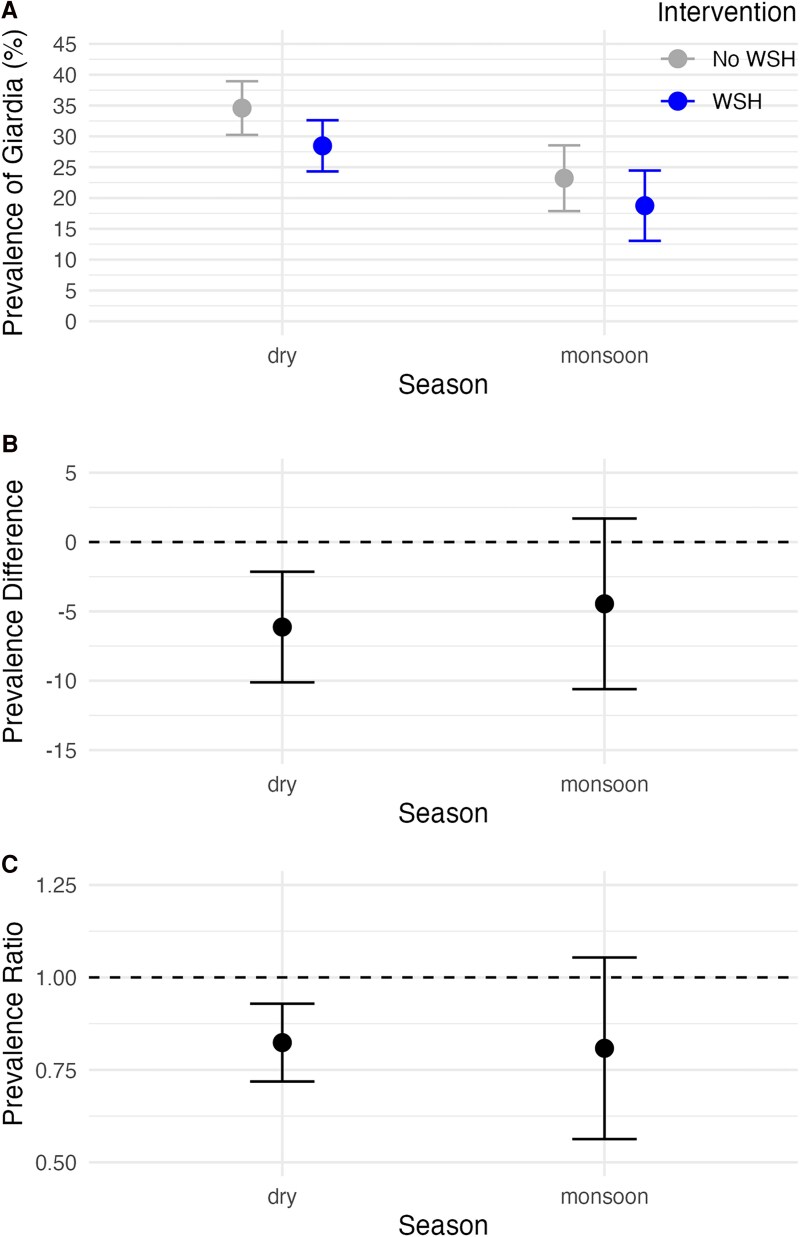
Prevalence of Giardia by season and their differences and ratios between intervention group. *A)* Giardia prevalence between the WSH and no WSH groups during the dry and monsoon seasons. The standard errors were estimated using linear combinations of the regression coefficients using the variance-covariance from the model. We accounted for clustering at block level using robust standard errors. *B)* Difference in Giardia prevalence and their 95% confidence interval (95% CI) between the intervention groups during the dry and monsoon seasons. *C)* Ratio of Giardia prevalence and their 95% CI between intervention groups by season.

Overall, *Giardia* prevalence was higher in the dry season (32%) than during to the monsoon season (21%). In both seasons, prevalence was lower among children in the WSH group than among those in the no WSH group. During the dry season, *Giardia* prevalence was 29%(95% confidence interval [95% CI]: 24% to 33%) in the WSH group and 35%(95% CI: 30% to 39%) in the no WSH group (Figure 2*A*). During the monsoon season, prevalence was 19% (95% CI: 13–25) in the WSH group and 23% (95% CI: 18–29) in the no WSH group.

The estimated absolute reduction in *Giardia* prevalence due to WSH was −6.1% (95% CI:, −10.1 to −2.1) during the dry season and −4.5% (95% CI: −10.6 to 1.7) during the monsoon season (Figure 2*B*). On the relative scale, the corresponding prevalence ratios were 0.82 (95% CI: 0.73–.94) and 0.81 (95% CI: 0.60–1.10), respectively (Figure 2*C*). Although the point estimate for the absolute reduction was slightly larger during the dry season, there was no statistical evidence of effect modification by season on either the additive scale (*P*-value for heterogeneity of .66) or the multiplicative scale (*P*-value for heterogeneity of .91). These results therefore lower *Giardia* prevalence among children in the WSH group in both seasons but provide limited evidence that the magnitude of the WSH effect differed by season.

### Effect Modification by Cumulative Exposure to Dry and Monsoon Seasons

**Figure 3.**
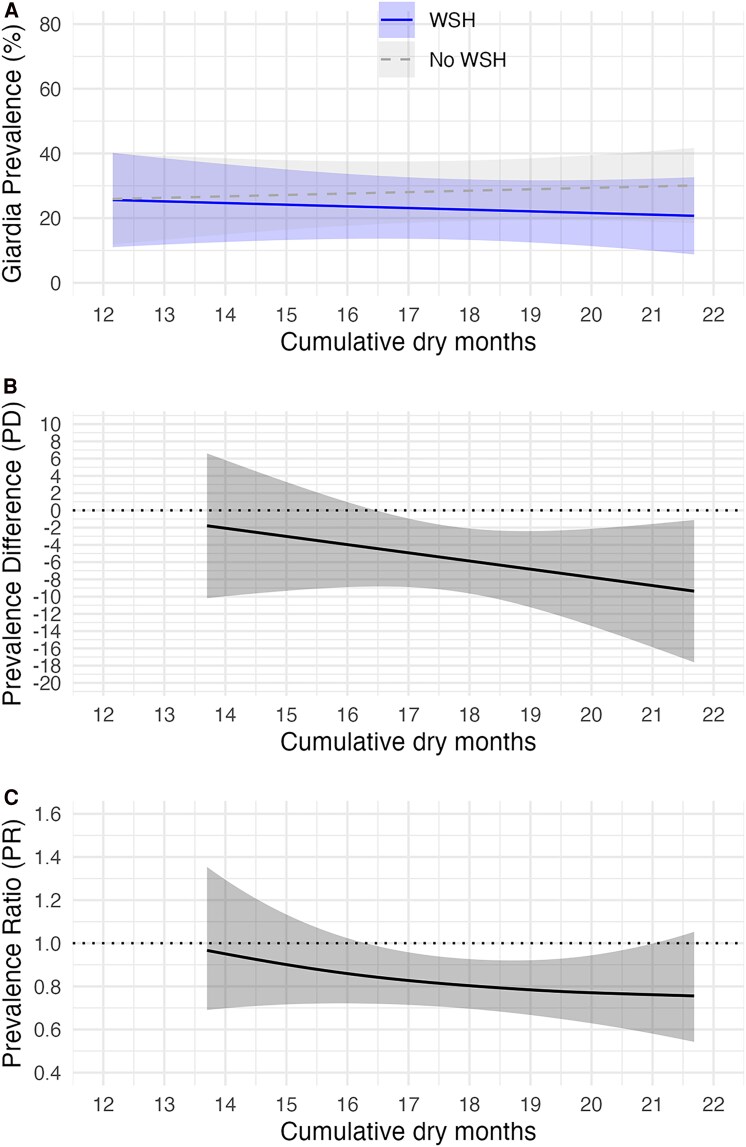
Prevalence of Giardia and their differences and ratios among children with varying exposure to dry months: A comparison between those receiving Water, Sanitation, and Handwashing (WSH) interventions and those without, within a factorial design. *A)* Giardia prevalence and their 95% confidence interval (95% CI) among children along the range of cumulative months exposed to dry season between the WSH and no WSH groups. *B)* Difference in Giardia prevalence and their 95% CI along the range of cumulative dry months. *C)* Ratio of Giardia prevalence and their 95% CI along the range of cumulative dry months. Adjusted for age, birth year, Nutrition and the interaction between dry months*WSH in the model. Used the tidymv package to estimate the smooth difference and their 95% CI along the range of dry months.

*Giardia* prevalence showed little change with cumulative months of dry season exposure among children who did not receive improved WSH. *Giardia* infection was generally lower among children who received improved WSH compared with those who did not across the range of dry season exposure (12.2 to 21.7 months), although the magnitude of the estimated difference varied across the exposure range with at least 17 months, uncertainty was greater at the lower and upper ends (Figure 3). When examining cumulative months of monsoon exposure, the effect of WSH was generally consistent across the range of monsoon months (Supplementary Fig. 1). The monsoon months are not simply the inverse of dry months as children's birth dates vary in relation to the timing of monsoon seasons.

### Additional and Sensitivity Analyses

We found that the Nutrition intervention had no effect on *Giardia* prevalence (Supplementary Fig. 2). When repeating the analysis excluding the first 6 months of life, the results were consistent with the main findings, albeit the months of exposure variable is shifted due to omitting the first 6 months (Supplementary Figs. 3 and Fig. 4). *Giardia* prevalence increased with greater cumulative exposure to dry months (Supplementary Fig. 4a), reaching up to 50% in the no WSH group by 20 months. The protective effect of WSH also increased with more exposure to dry months (Supplementary Fig. 4).

## DISCUSSION

We found that *Giardia* risk varied seasonally and was lower among children who received WSH compared with those who did not. The relative reduction due to WSH was similar in the dry and monsoon seasons, whereas the absolute reduction was slightly larger in the dry season, where overall *Giardia* prevalence was higher. However, interaction tests did not provide evidence that season modified the effect of WSH on either the additive or multiplicative scale.

We also show that children experienced different cumulative dry and monsoon seasons periods depending on their timing of birth, resulting in heterogeneous exposures to seasonal conditions from birth to the time of measurement. In analysis of cumulative seasonal exposure, *Giardia* prevalence showed little change with increasing dry season exposure among children who did not receive WSH, whereas prevalence was generally lower among children who received WSH across much of the observed exposure range. Together, the results show that improved household WSH consistently reduces the risk of *Giardia* infection, even in the context of large, seasonal increases in risk.

We highlight the value of leveraging birth timing and cumulative seasonal exposures when evaluating intervention effects. Children's exposure to dry and monsoon seasons varied and depended on their timing of birth, age, and measurement period, allowing us to link each child's infection outcome to their unique seasonal exposure history. This approach revealed that *Giardia* infection risk varied seasonally and that children with greater cumulative dry-month exposure appeared to have slightly larger absolute reductions in infection with improved WSH. These results suggest that sustained WSH improvements can provide consistent reductions in *Giardia* risk even with seasonally varying increases in risk.

This framework estimates children's cumulative exposure to climate conditions from birth to the time of measurement, providing a simplified representation of both the timing and duration of environmental conditions influencing infection risk. This analysis evaluates whether WSH-associated reductions in *Giardia* prevalence differed across children with different cumulative seasonal exposure histories. Increasing risk observed with longer dry season exposure among children without improved WSH suggests that prolonged exposure to dry conditions may facilitate sustained transmission and accumulation of infections over time.

When excluding the first 6 months of life of a child, a period when maternal antibodies may protect from infection [28], *Giardia* prevalence increased more with greater exposure to dry months (Supplementary Fig. 4a). WSH intervention during the dry months of the first 6 months of life did not yield big effects. We also observed that the protective effect of WSH also appeared earlier, from around 13 months of cumulative dry-season exposure suggesting that children may benefit more from the intervention once maternal immunity has declined, which may be due to higher *Giardia* infection and more potential to benefit from the intervention [29].

Our findings are consistent with previous studies using the WASH Benefits Bangladesh data showing that the rainy season was associated with lower *Giardia* prevalence [9]. This pattern suggests that rainfall may not be the primary driver of *Giardia* transmission in this setting. Alternative pathways, including person-to-person transmission, environmental contamination of soil or household surfaces, contamination of limited or shared water sources during dry periods, reduced dilution of fecal material, and young children's contact with contaminated environments, may contribute to dry-season transmission. In addition, we demonstrate consistent benefits of improved WSH and no significant benefit of the Nutrition intervention in reducing *Giardia* [5]. Findings from other settings suggest that the association between *Giardia* and environmental factors varies across contexts. A systematic review reported mixed evidence [8]. Some studies found that cyst counts increased in warm and moist conditions, whereas others suggested that dry or low-humidity environments may facilitate transmission [8]. A meta-analysis examining WSH interventions reported that the protective effects on diarrhoea were stronger during the dry season [30]. This aligns with our findings that WSH interventions may have the greatest impact during the dry period. However, our findings contradict with a previous analysis using the same data showing greater diarrhea reductions during wet seasons [13, 14] highlighting that the direction of seasonal effects may depend on the etiological agent (eg, all cause diarrhea vs bacteria vs protozoa vs virus). The slightly larger absolute reduction observed during the dry season may reflect the higher underlying *Giardia* prevalence rather than greater intervention efficacy. Higher baseline prevalence provides greater opportunity to detect absolute differences, whereas relative effects were similar across seasons and formal interaction tests provided no strong evidence of effect modification. Both heavy rainfall and prolonged dry periods can influence enteric pathogen transmission, albeit through distinct pathways. Intense precipitation or monsoon rains may overwhelm sanitation and drainage infrastructure, mobilize animal and agricultural waste into surface water used for recreation or irrigation, and contaminate groundwater sources [31, 32]. Rainfall also facilitates the transport of pathogens across environmental compartments. Conversely, extended dry conditions and extreme temperature can elevate infection risk by concentrating pathogens in diminishing or stagnant water sources, reducing water availability for hygiene, and increasing reliance on contaminated supplies [33]. Drought conditions can lead to the accumulation of enteric pathogens in surface waters, which may subsequently be flushed and diluted during rainfall events [8, 34]. Consistent with this mechanism, *Cryptosporidium* oocyst concentrations have been shown to correlate negatively with precipitation, not because rainfall introduced additional oocysts, but because it diluted those already present [8, 35]. A similar process may underlie *Giardia* transmission, whereby pathogen concentrations and exposure risk rise during dry periods and decline with increased rainfall and water turnover during the monsoon. In our study, this mechanism may be reflected in the observed decrease in *Giardia* prevalence with greater cumulative exposure to monsoon months (Supplementary Fig. 2a) and the corresponding increase with longer exposure to dry months (Figure 3*A*) in the no WSH group. These findings suggest that improved WSH conditions may provide resilience during dry periods, when concentrated contamination and reduced water availability elevate transmission risk.

Our study had several limitations. First, the trial was conducted in areas that were not highly prone to flooding during the seasonal monsoon, which may have led to underestimation or overestimation of the effects of WSH on *Giardia* compared with outcomes that might be observed in more flood-prone settings. Second, the study only measured children ages 22 to 38 months at the time of measurement and so did not characterize risk at other ages. Third, we defined season using only the monsoon periods of 2015–2016, which may not fully reflect broader climate variability or interannual fluctuations. To address this, we also created a continuous measure of cumulative exposure to dry and monsoon months from birth to measurement, which may better capture these dynamics. Future studies incorporating serologic measures, such as Giardia-specific immunoglobulin G responses could provide deeper insight into effect heterogeneity across pathogens and seasonally varying conditions. This may be particularly useful for *Giardia*, which is often asymptomatic, because antibody responses may capture evidence of prior exposure and complement PCR measures that primarily reflect current infection or shedding.

The study also had several strengths. We analyzed the effect of WSH on *Giardia* within a 2 × 2 factorial design which enhanced statistical power [21]. In addition, we incorporated cumulative exposure to dry and monsoon seasons providing a proxy for cumulative seasonal exposure history while also accounting for climate-related heterogeneity.

In conclusion, we demonstrate that *Giardia* risk varies seasonally, with improved WSH providing consistent reduction in risk even during period of elevated infection risk. This finding underscores the importance of WSH interventions for strengthening population resilience to seasonally and climate-driven fluctuations in disease transmission, particularly in climate-sensitive and low-resource settings.

## Supplementary Material

ofag430_Supplementary_Data

## Supplementary Data

Supplementary materials are available at *Open Forum Infectious Diseases* online. Consisting of data provided by the authors to benefit the reader, the posted materials are not copyedited and are the sole responsibility of the authors, so questions or comments should be addressed to the corresponding author.
