# Supplementary material for: Seasonally Varying Effects of Improved Water, Sanitation, and Handwashing Interventions on Giardia Infection in Bangladesh
Source: Open Forum Infect Dis. 2026 Jul 16;13(8):ofag430. doi: 10.1093/ofid/ofag430 (PMC13430655; doi:10.1093/ofid/ofag430)
Supplement: ofag430_Supplementary_Data [file ofag430_supplementary_data.docx]

**Supplementary Material to**

**Seasonally varying effects of improved water, sanitation and handwashing interventions on Giardia infection in Bangladesh**

Pearl Anne Ante-Testard^1^*****, Francois Rerolle^1^, Mahbubur Rahman^2^, Rashidul Haque^2^, Shimul Das^2^, Sarker Masud Parvez^2,3^, Ayse Ercumen^4^, Audrie Lin^5^, Stephen P. Luby^6^, Tarik Benmarhnia^7,8^, Benjamin F. Arnold^1^

1. Francis I. Proctor Foundation and the Department of Ophthalmology, University of California, San Francisco, San Francisco, CA, USA
2. International Centre for Diarrhoeal Disease Research, Dhaka, Bangladesh
3. School of Public Health and Social Work, Queensland University of Technology, Brisbane, Australia
4. Department of Forestry and Environmental Resources, North Carolina State University, Raleigh, NC, USA
5. Department of Microbiology and Environmental Toxicology, UC Santa Cruz, Santa Cruz, CA, USA
6. Division of Infectious Diseases and Geographic Medicine, Stanford University, Stanford, CA, USA
7. Scripps Institution of Oceanography, University of California, San Diego, 8885 Biological Grade, La Jolla, CA 92037, USA
8. Irset Institut de Recherche en Santé, Environnement et Travail, UMR-S 1085, Inserm, University of Rennes, EHESP, Rennes, France

***Corresponding author:** Pearl Anne Ante-Testard; Francis I. Proctor Foundation and the Department of Ophthalmology, University of California, San Francisco, 490 Illinois St., Floor 2, San Francisco, CA 94158, USA; [pearl.ante@ucsf.edu](mailto:pearl.ante@ucsf.edu).

**Supplementary Fig. S1**. **Prevalence of Giardia among children with varying exposure to monsoon months: A comparison between those receiving water, sanitation, and handwashing (WSH) interventions and those without, within a factorial design.** Adjusted for age, birth year, Nutrition and the interaction between monsoon months and WSH in the model.


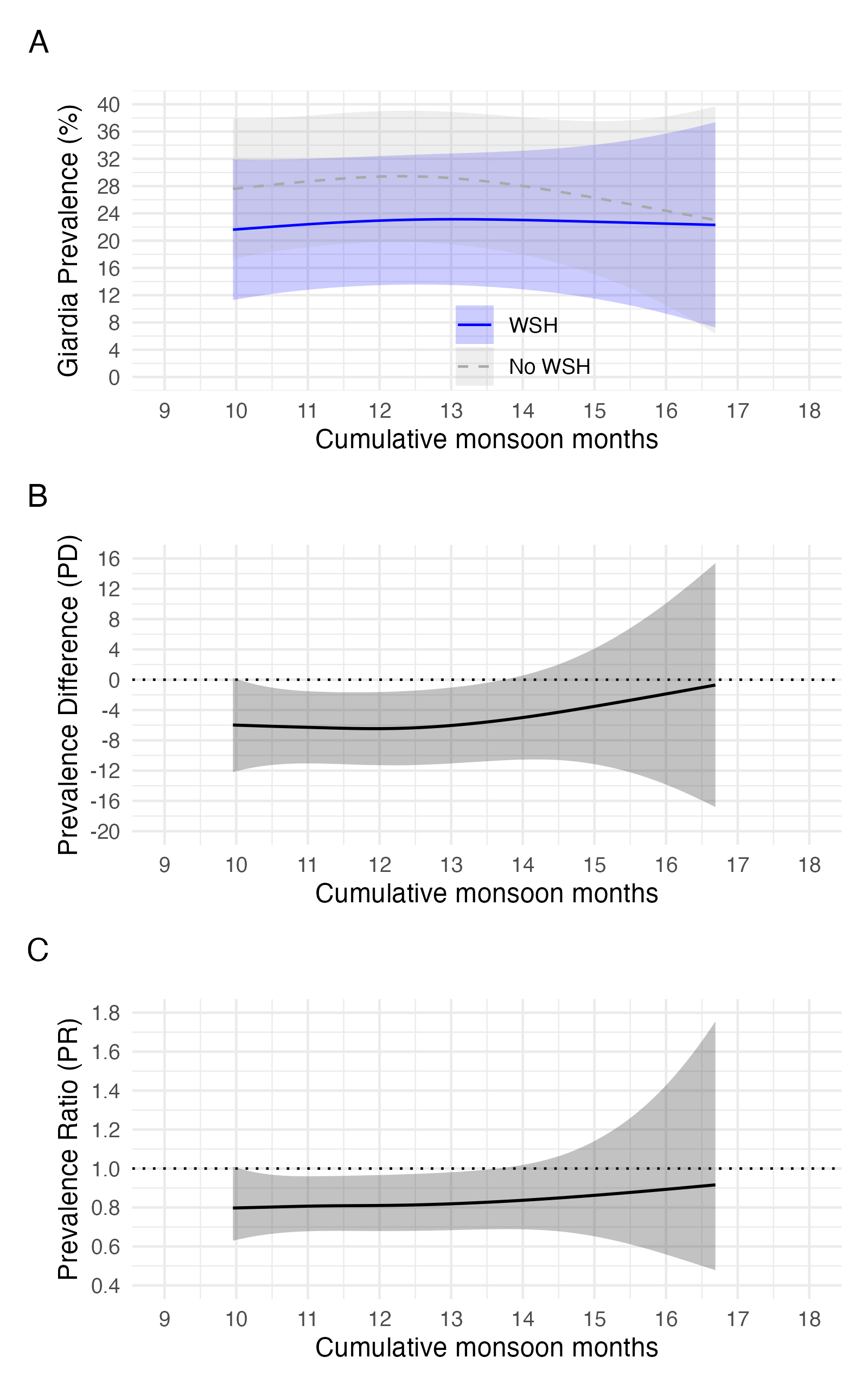


**Supplementary Fig. S2**. **Prevalence of Giardia among children with varying exposure to dry months: A comparison between those receiving nutrition interventions and those without, within a factorial design.** Adjusted for age, birth year, water, sanitation and handwashing (WSH) group and the interaction between dry months and Nutrition in the model.

**
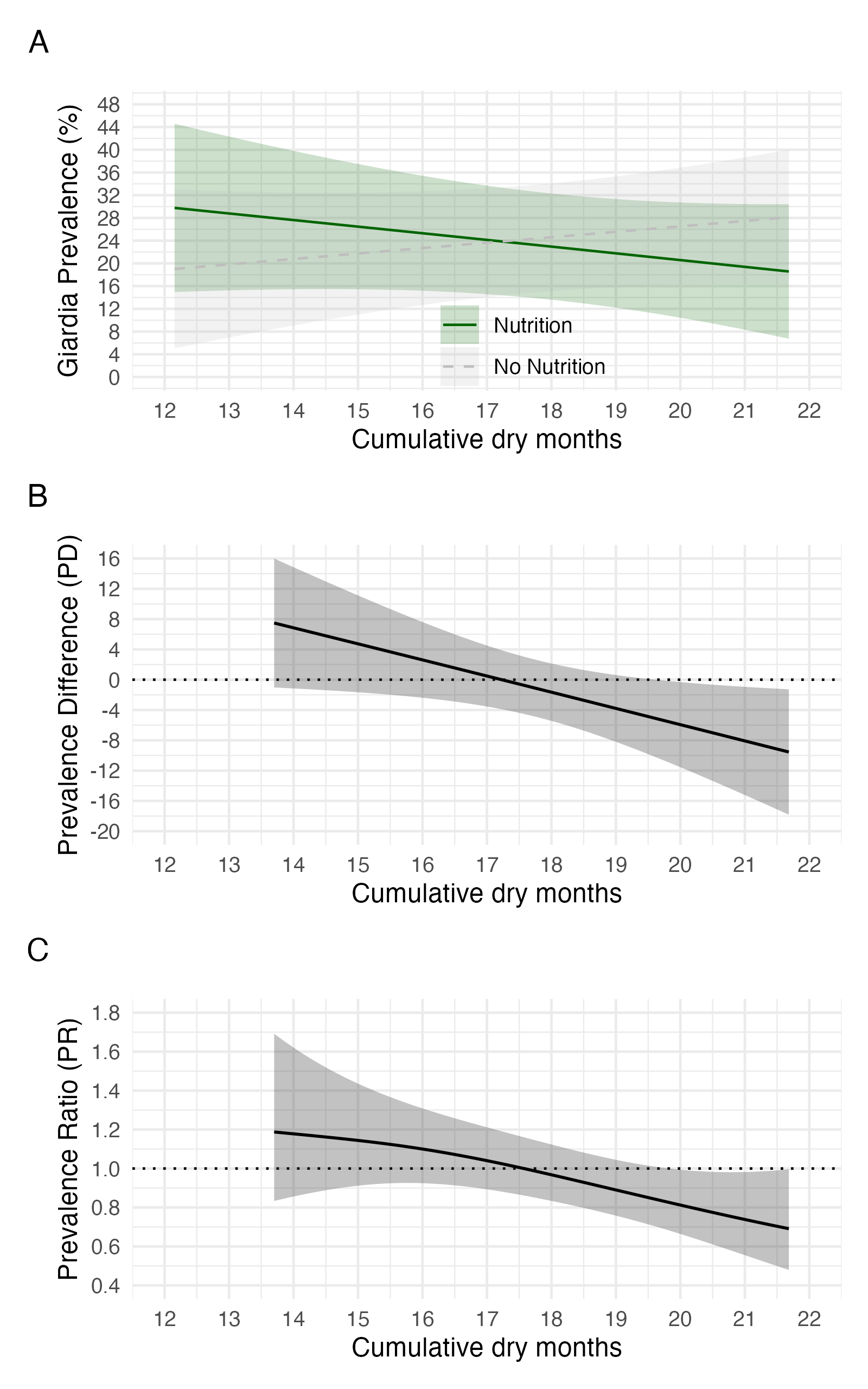
**

**Supplementary Fig. S3. Children in the cohort experienced the dry and monsoon seasons from six months after birth to the time of measurement. A.** Six months after birth of children included in the study with monsoon months shaded in grey. **B.** Distribution of the number of dry months by study arms. **C.** Timeline from six months after birth to measurement, with monsoon months shaded in grey to show the cumulative months of exposure to monsoon. Children were ordered by birth date.

**
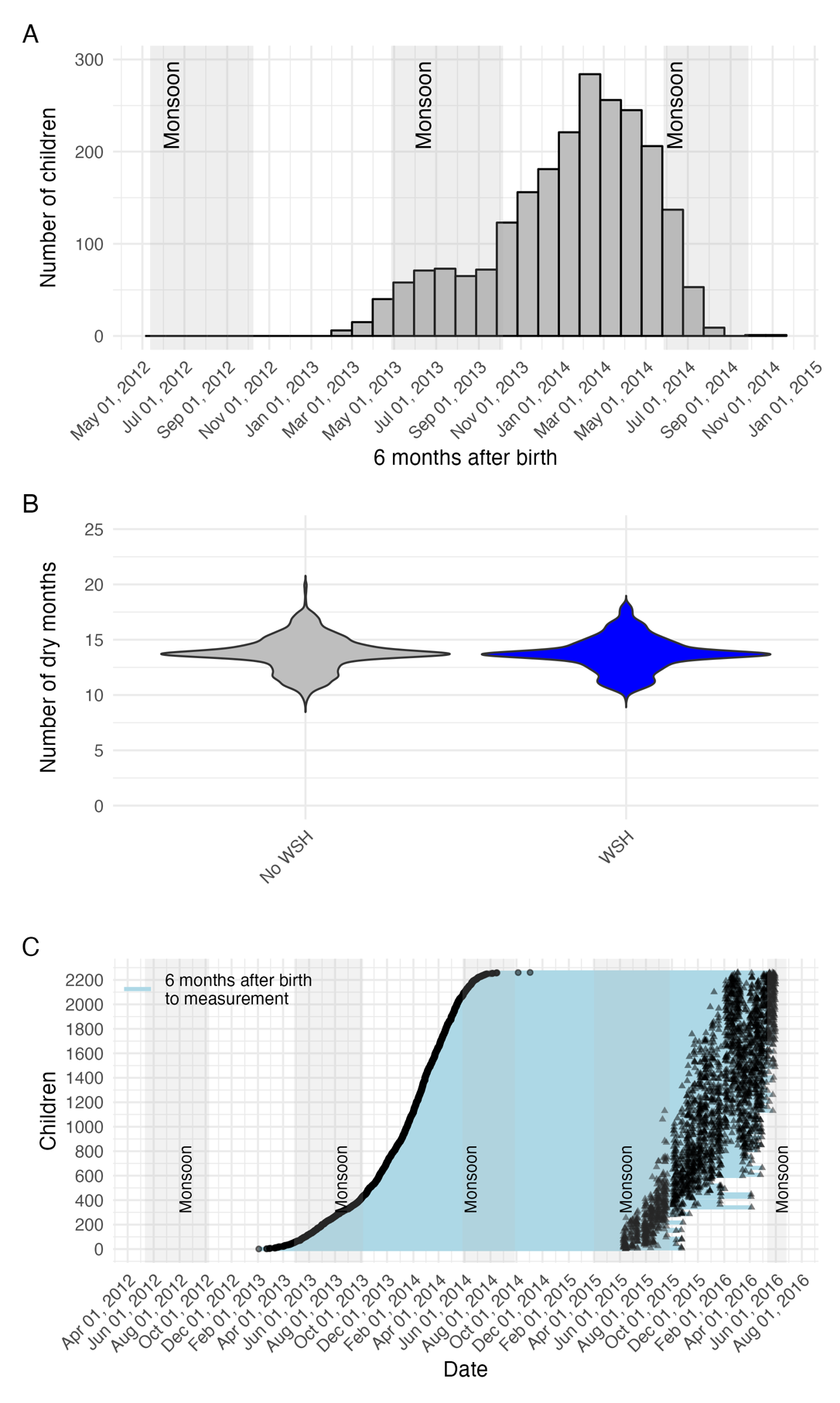
**

**Supplementary Fig. S4**. **Sensitivity analysis excluding the first 6 months of life to estimate the number of dry months a child had experienced.** Prevalence of Giardia among children with varying exposure to dry months: A comparison between those receiving water, sanitation, and handwashing (WSH) and those without, within a factorial design. Adjusted for age, birth year, Nutrition intervention and the interaction between dry months and WSH in the model.

**
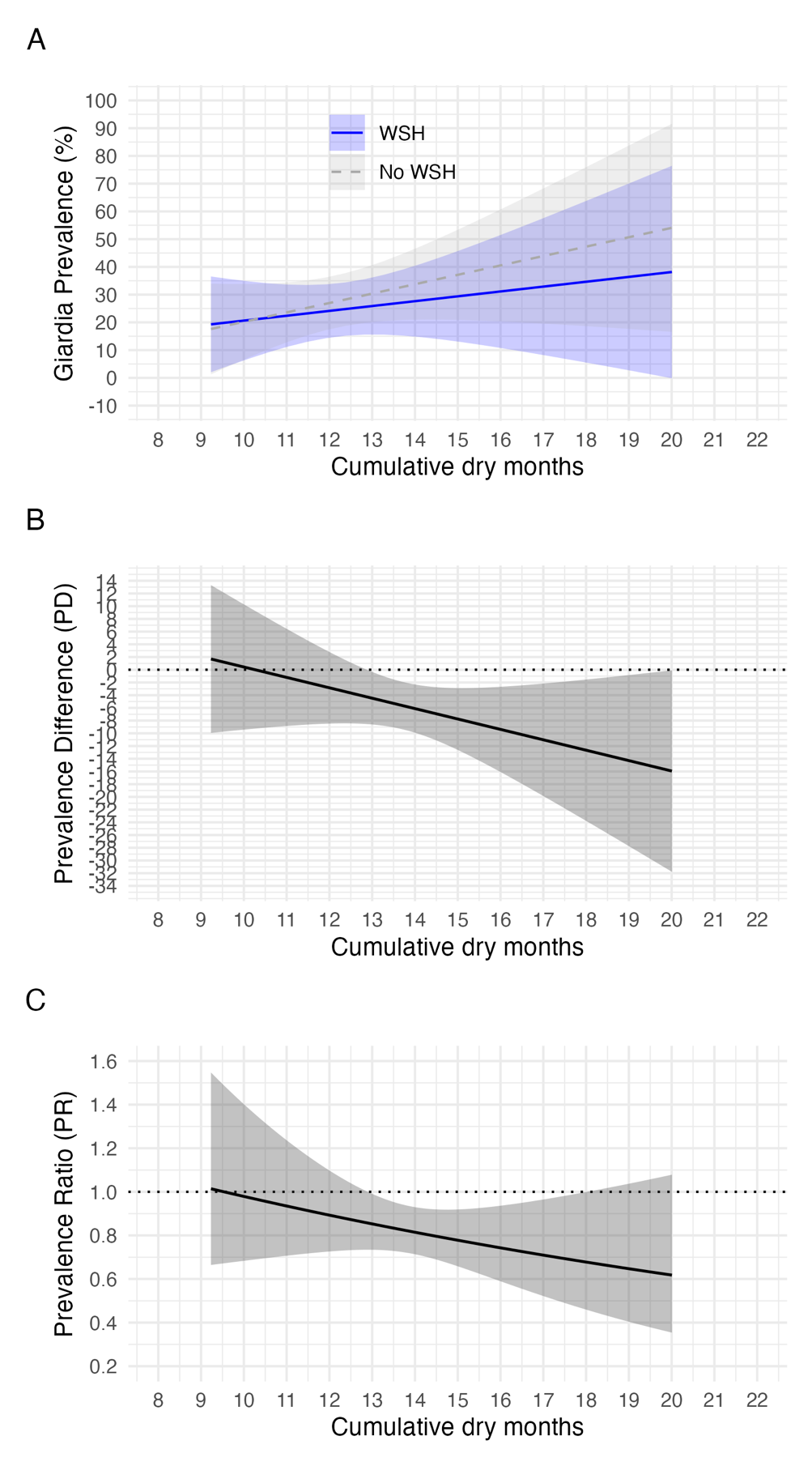
**
